# District health managers perspectives of introducing a new service: a qualitative study of the community-based newborn care programme in Ethiopia

**DOI:** 10.1186/s12913-021-06792-8

**Published:** 2021-08-09

**Authors:** Della Berhanu, Iram Hashmi, Joanna Schellenberg, Bilal Avan

**Affiliations:** 1 London School of Hygiene & Tropical Medicine (LSHTM), London, WC1E 7HT UK; 2 Ethiopian Public Health Institute, Addis Ababa, Ethiopia

**Keywords:** District health managers, Neonatal sepsis, Newborn health, Implementation, Community health workers, Non-governmental organizations

## Abstract

**Background:**

The planning, resourcing, implementation and monitoring of new programmes by district health managers is integral for success and sustainability. Ethiopia introduced the Community-Based Newborn Care programme in 2014 to improve newborn survival: an innovative component allowed community health workers to provide antibiotics for young infants with possible serious bacterial infection when referral was not possible. Informed by the World Health Organization health system building block framework, we aimed to study the capacity and operational challenges of introducing this new health service from the perspective of programme implementers and managers at the district level 20 months after programme initiation.

**Methods:**

This qualitative study was part of a programme evaluation. From November to December of 2015, we conducted 28 semi-structured interviews with staff at district health offices, health centres and implementing Non-Governmental Organisations in 15 districts of four regions of Ethiopia. Verbatim transcripts were analysed using a priori and emerging themes.

**Results:**

In line with the government's commitment to treat sick newborns close to their homes, participants reported that community health workers had been successfully trained to provide injectable antibiotics. However, the Community-Based Newborn Care programme was scaled up without allowing the health system to adapt to programme needs. There were inadequate processes and standards to ensure consistent availability of (1) trained staff for technical supervision, (2) antibiotics and (3) monitoring data specific to the programme. Furthermore, Non-Governmental Organizations played a central implementing role, which had implications for the long-term district level ownership and thus for the sustainability of the programme.

**Conclusion:**

In settings where sustainable local implementation depends on district-level health teams, new programmes should assess health system preparedness to absorb the service, and plan accordingly. Our findings can inform policy makers and implementers about the pre-conditions for a health system to introduce similar services and maximize long-term success.

**Supplementary Information:**

The online version contains supplementary material available at 10.1186/s12913-021-06792-8.

## Background

One of the sustainable development goals is to reduce neonatal mortality to 12 deaths per 1000 live births by 2030 [1]. While Ethiopia successfully reduced under-five mortality, between 2011 and 2016 neonatal mortality only decreased from 37 to 29 deaths per 1000 live births [2–4]. Low-income countries with high neonatal mortality have bottlenecks in the health system building blocks framed by the World Health Organization (WHO): governance, health workforce, service delivery, health management information systems, essential medicines and financing [5–7].

In many countries, the district health system plays a major role in the provision of newborn health services. Constraints within the district health system along the WHO building blocks can lead to operational and capacity related challenges that affect the provision of high-quality community based newborn care services. As such, there is a need for a well-functioning district health system for planning and implementing health services [8].

In view of Ethiopia’s high neonatal mortality, starting in 2014, the Community-Based Newborn Care programme (CBNC) was nationally implemented in three phases, through the district health system [9]. It aimed to improve antenatal, intrapartum and newborn care by strengthening: 1) the Health Extension Programme, which is the platform for primary health care service delivery, and 2) the primary health care unit, which is comprised of five health posts, their referral health centre and a primary hospital [10]. Furthermore, given that infections along with asphyxia and prematurity were the main causes of neonatal mortality, the unique feature of this initiative was shifting the task of managing sick young infants (0–59 days old) with any danger sign of possible serious bacterial infection from clinicians at health centres and hospitals to community health care workers [11, 12]. Young infants whose caregivers were unable or refused to go to higher levels of care could be provided with intramuscular gentamicin and oral amoxicillin dispersible tablets for 7 days by Health Extension Workers (HEWs) at health posts or through outreach services. This treatment protocol was based on global evidence that trained and supported community health care workers can identify and treat newborns with possible serious bacterial infections [13]. Locally, between 2008 and 2013, the cluster-randomized Community-Based Intervention for Newborns in Ethiopia trial showed that, despite inadequate service utilization, HEWs were able to deliver outpatient antibiotic treatment of newborns with possible serious bacterial infection [14].

The CBNC programme was a large-scale government lead initiative that was scaled-up to the whole country over the course of 6 years. Lessons from such large-scale initiatives, with evidence on the successes and challenges of implementing and integrating community-based services into existing health systems, can help to optimise future programmes [10]. The WHO health system building blocks provide a framework for describing the context within which such health services are delivered. This study used these building blocks to assess the challenges of introducing the management of possible serious bacterial infection through the CBNC programme into the district health system, 20 months after the programme initiation. We describe how the programme was implemented, identify district-level operational and capacity challenges, and present participant recommendations to improve the programme. Such information can provide invaluable insights for those planning to integrate a new primary care service into a district health system.

## Methods

### Study context

**Fig. 1:**
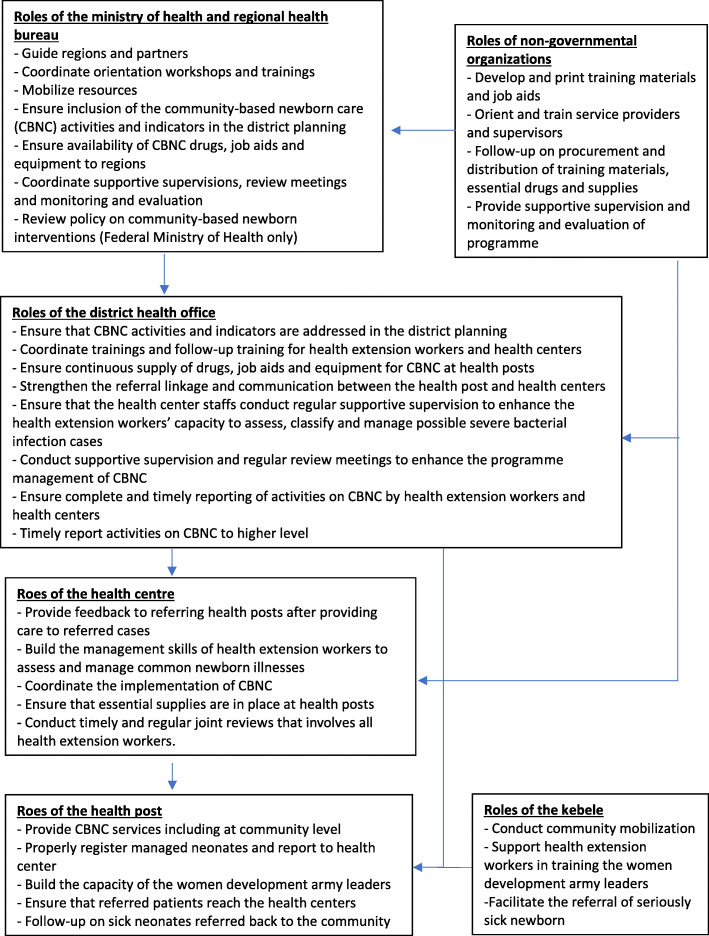
The Community-Based Newborn care programme implementers roles and responsibilities across the different layers of the health system

Ethiopia has a three-tier health care delivery system [10]. At the tertiary level, a specialized hospital serves 3.5–5 million people. At the secondary level, a general hospital serves 1–1.5 million people. At the primary level, five primary health care units, each comprised of five satellite health posts and one health centre, and their referral primary hospital serve approximately 125,000 people. Each health post typically has two HEWs who serve a whole kebele (village) with an approximate population of 5000. Health posts are supervised by the kebele administration and district health office and receive technical support from health centres. HEWs are assisted by a volunteer group of women known as the Women’s Development Army. The Woreda (district) Health Office is the health administrative body at the district level, whose responsibility is limited to primary health care services [15]. Districts have their own budget, which is annually divided among the different sectors, including health. Planning for primary health care services at the district takes place annually with the aim of meeting local health needs within the context of national targets [16]. The district health office also oversees the logistics of supplies and drugs, provides supervision to health centres and health posts, and monitors data on health service provision [15]. Figure 1 shows the different levels of the health system along with the roles and relationships of the various CBNC programme implementers [17, 18].

### Description and rollout of the CBNC Programme

**Fig. 2:**
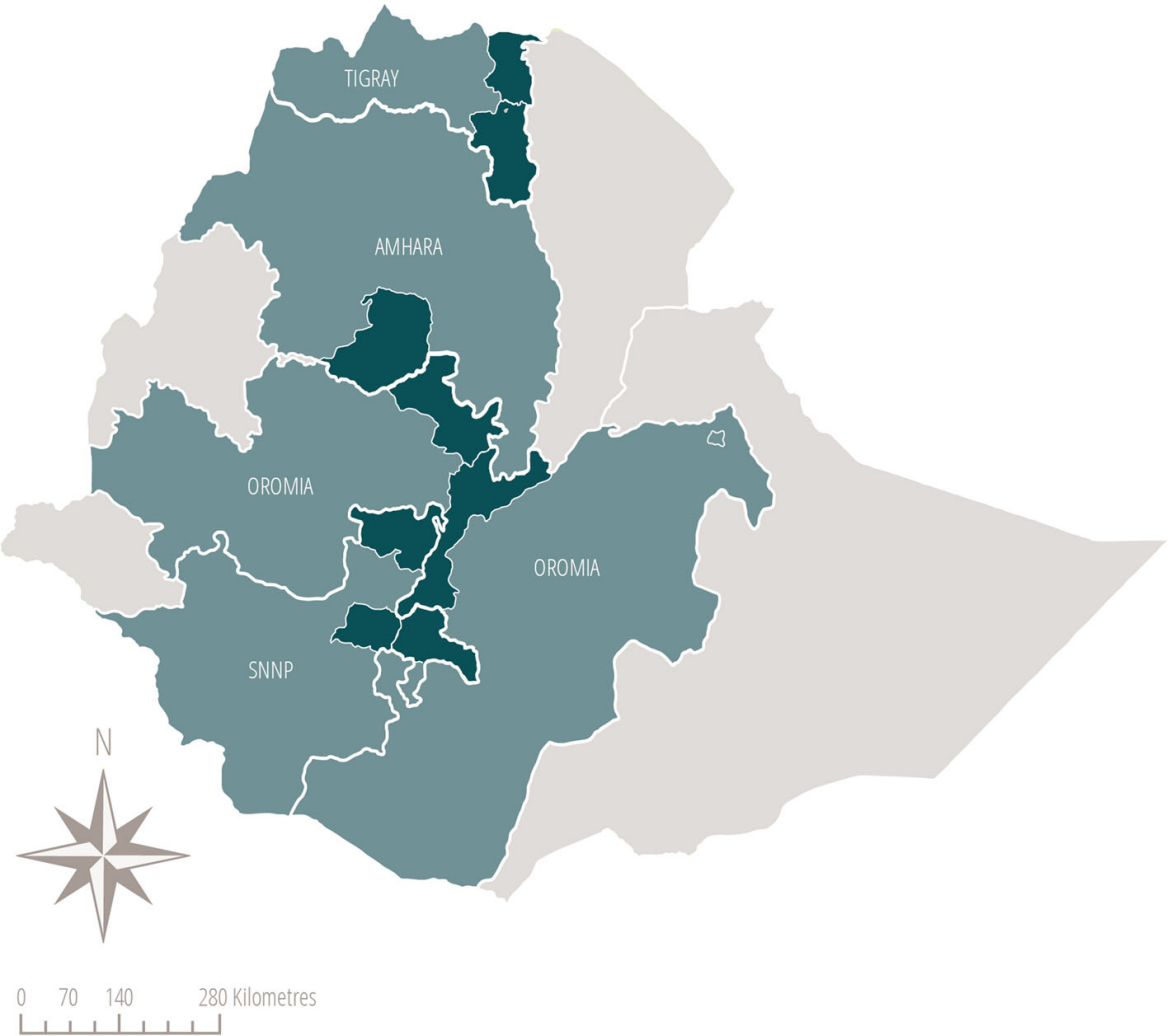
The three phases of Community-Based Newborn Care programme implementation: phase 1 (March 2014) shown in dark green, phase 2 (January 2015) in pale green and phase 3 shown in grey (January 2018). Map produced by the authors

The CBNC programme had nine components enabling HEWs to provide services across the continuum of care: 1) early identification of pregnancy, 2) focused antenatal care, 3) promotion of facility delivery, 4) safe and clean delivery, 5) immediate newborn care 6) recognition and management of asphyxia, 7) prevention and management of hypothermia, 8) management of preterm and low birth weight babies, and 9) management of possible serious bacterial infection with antibiotics when referral was not possible. The 9th component, the management of possible serious bacterial infection with antibiotics when referral was not possible, was new to the Health Extension Programme. In March 2014, as part of phase 1, the CBNC programme was launched in 104 districts across four regions (Amhara, Tigray, Oromia, and Southern Nations, Nationalities and Peoples (SNNP)) of Ethiopia as a learning phase to inform the scale-up process for the rest of the country. In January 2015, as part of phase 2 of the implementation, the CBNC programme was initiated in the other districts of the four regions. In January 2018, as part of phase 3, the programme was launched in the remaining districts of the country (Fig. 2). The Federal Ministry of Health implemented the CBNC programme in partnership with Save the Children, Last 10 Kilometres/John Snow Inc. and Integrated Family Health Programme, with support from UNICEF.

### Study setting

This study was part of the evaluation of the government lead CBNC programme. The London School of Hygiene and Tropical Medicine, in collaboration with JaRco Consulting, were commissioned by the Ethiopian government to undertake the evaluation of CBNC. JaRco consulting is an international development consulting company based in Ethiopia and was The School’s measurement, learning and evaluation partner. The qualitative interviews were conducted in 12 phase 1 and phase 2 districts across Amhara, Tigray, Oromia, and Southern Nations, Nationalities and Peoples regions of Ethiopia. At the time of the study, phase 1 districts had approximately 14 months of the CBNC programme implementation, and phase 2 areas had approximately 20 months of implementation.

### Study design

This study was part of the CBNC programme evaluation [19]. The overall evaluation included baseline (2013) and endline surveys (2017) at the household and health facility level, as well as a midline assessment focusing on the health facility level. Two rounds of qualitative data were collected, in 2014 and 2015: the latter is the source of data for the current study.

### Study participants

This study was conducted from November to December of 2015. From each study district we planned to include one health centre staff and one district health office staff trained in the CBNC programme. If trained staff were not available, individuals responsible for overseeing the CBNC programme for the previous 3 months were interviewed. The study also planned to include staff from Non-Governmental Organizations (NGOs) that worked in partnership with the Federal Ministry of Health to implement the CBNC programme.

### Data collection and analysis

Data were collected through semi-structured in-depth interviews. The interview guide was translated from English into Amharic, Oromifa, Tigrigna and was informed by the study team’s previous work on the evaluation of the CBNC programme [9]. The guide was further refined through formative interviews with the district, health centre and NGO staff members. The interview guide is provided in Additional file 1. Data collectors were trained on the study aims, selection of study participants and interview guides. We aimed to stop collecting data when information saturation was reached.

Interviews were captured through a tape recorder and written notes. Verbatim transcripts were then translated into English.

We conducted a thematic analysis. The transcripts were read multiple times to ensure familiarity with the data. We used five out of the six WHO health system building blocks as general themes to categorize the CBNC programme implementation, associated challenges and recommendations to improve programme implementation: the health workforce for the CBNC programme (training, supervision and performance review and clinical mentoring); access to essential medicines; the health information system (reporting); service delivery (referrals); and, governance and ownership which are key features of leadership at the district level that were crosscutting across the other four health system building blocks. Within each theme the transcripts were coded using a priori and emerging codes and sub-codes. The a priori codes and sub-codes are provided in Additional file 2. The data were ranked based on the frequency of occurrence.

## Results

A total of 28 out of the planned 36 interviews were conducted, comprised of 10 health centre staff, 9 district health office and 9 NGO staff. One district health office had no CBNC trained staff nor an individual assigned to oversee the programme. Two districts were supported by the same NGO and we interviewed one staff member who could speak on behalf of both districts. The remaining interviews were not conducted due to information saturation.

A majority of participants (*n* = 21) had a minimum educational qualification of a Bachelor’s degree in nursing, four had a Diploma in nursing and three had a Master of Science degree. Exactly half of the participants had been in their current position for at least 2 years. CBNC programme training was provided for 21 out of the 28 study participants. Seven participants from the district health offices and health centres had not received training, but instead had experience managing the services for 3 months or longer.

In the rest of the results section, for each of the five previously stated WHO health system building blocks, we present the mechanisms used to implement the CBNC programme at the district level, as well as the identified challenges and recommendations to improve the programme. One block, financing, was not covered as the budget for the CBNC programme was dealt with at the federal level by the Ministry of Health and implementing partners.

### Health workforce for the CBNC programme: training, supervision and performance review and clinical mentoring meeting

#### Mechanisms for training staff for the CBNC programme

Participants said the CBNC programme training lasted 4–5 days with 20–25 participants per training session. Training included all nine components of the CBNC programme, with a focus on practical rather than theoretical learning. At each primary health care unit, selected health centre and district health office staff were trained alongside HEWs by trained staff from zones and regions. Participants also stated that the training included components on referral, reporting and record keeping.

*“Trainees were well-trained on the identification of newborns, identification of sick newborns, diagnosing and identifying diseases in sick newborns and giving appropriate treatment”* (NGO – SNNP)

Although the majority of participants felt very optimistic about the HEWs’ skills in the management of sick young infants after receiving CBNC programme training, they explained several training related challenges detailed below.

#### Challenges in training staff for the CBNC programme

##### Insufficient days for training

Nearly half of the study participants considered the 5-day training for HEWs was insufficient to cover all practical aspects of the CBNC programme. Particularly, activities that could be conducted by HEWs that could potentially lead to an increase in utilization of services for newborn was said to be lacking.

*“In order to improve HEWs capacity in demand creation there was a need to demonstrate these demand creation activities on the field exercise conducted during the training. However, in the CBNC training, the role of HEWs in demand creation was overlooked.”* (District health office staff – Amhara)

##### Insufficient and inadequate training for the CBNC programme support staff

Many participants highlighted that insufficient district health office and health centre staff members were trained to fulfil the administrative responsibility for the CBNC programme. Two participants from NGOs also showed concerns over the high staff turnover requiring continuous training for new staff.

#### Mechanisms for supervising service providers for the CBNC programme

With the start of the CBNC programme, it was assumed that health centre staff would provide weekly supportive supervision to health posts. Staff from district health offices and health centres received additional training on how to provide supportive supervision and assess HEWs’ performance on the CBNC programme related activities. During supervisory visits, health centre staff reported assessing the HEWs’ performance using a checklist. Nearly all study participants explained that supervision included a direct observation of care provided to young infants by HEWs, home visits, discussions and technical support. Constructive written feedback was also given to HEWs. A health centre staff member explained,

*“From the neonatal registration book, we cross check the coherence of name, treatment and follow up given. We also assess the registration book for completeness of relevant information”* (Health centre staff – Tigray)

Supportive supervision was considered an excellent mechanism for delivering regular technical support to HEWs and enhancing their capacity. HEWs were reported to have shown considerable improvement in treating possible serious bacterial infection in young infants because of regular support and supervision by trained staff from health centres and district health offices.

#### Challenges of providing CBNC programme specific supervision for service providers

##### Insufficient and inadequate staff for the CBNC programme supervision

HEWs were said to not receive the much-needed programme-specific supportive supervision because not enough health centre staff had been trained in the CBNC programme. Despite the expectation of weekly visits, two-thirds of the study participants expressed that the CBNC programme specific supervision was done monthly by either trained district, health centre or NGO staff. Health centre and district health office staff also indicated that the CBNC programme lacked depth on how to provide supportive supervision for HEWs and assess their technical skills, which affected the quality of support that they provided.

##### Inconsistencies in supervision processes

While more than half of the participants stated that supportive supervision included a CBNC programme checklist, participants from district health offices and health centres indicated that supervision was done with no particular focus on the programme. One of them described:

*“We don’t have a standard checklist, rather we use a checklist prepared by ourselves.”* (Health centre staff – Amhara)

##### Issues of transportation

Transportation was considered a big challenge as supervisors had to travel long distances or to hard-to-reach areas resulting in less frequent supervisions.

##### HEWs unavailability

HEWs not being available at health posts and having a high workload was said to hinder supervision. HEWs engagement with other aspects of their work prevented timely and regular supervision,

*“It is a polio vaccination campaign. Of course, it is mandatory to conduct the polio campaign, you can do nothing about it … .in such case we will interrupt our supervision.”* (NGO-Amhara)

#### Mechanisms for providing a CBNC programme performance review and clinical mentoring meetings for service providers

Performance review and clinical mentoring meetings aimed to provide further support to HEWs. On average HEWs from 10 health posts and staff from health centres, the district health office and implementing NGO were expected to attend the performance review and clinical mentoring meeting. One interviewee described the participants list as follows:

*“The participants were HEWs, Woreda health office CBNC focal person, Woreda director, Save the children staff, Zone office staff, Woreda maternal and child health focal person, health centre director and health centre CBNC focal person.”* (NGO-Oromia)

The meetings reinforced HEWs’ management skills for young infants with possible serious bacterial infection. HEWs are expected bring their register books to the meetings, which was then reviewed to assess: if HEWs classified the illness of a young infant correctly given the recorded set of signs; given the recorded classification if they provided the appropriate treatment; and follow-up and outcome of the treated child. HEWs then receive clinical mentoring and coaching based on the identified skills gap. A collective visit to a household with a newborn is also conducted. Furthermore, participants discuss bottlenecks for service provision.

*“Then they will work in groups to identify limitations on the service provision using the registration books and their own experience. We will be there to support this exercise and give our own reflection on the discussions underway by different groups. They will raise the issues, underlying causes behind these problems and suggest the solution to overcome these problems”* (NGO-Amhara)

Woreda officials also collect important data to inform progress and quality of the programme. This two-day performance review and clinical mentoring meeting is held biannually.

The performance review and clinical mentoring meeting when it took place was said to create an opportunity to enhance the capacity and moral of the HEWs. It provided participants with an opportunity to learn from gaps that had been identified, which in turn could improve their performance. HEWs were said to benefit from sharing their experience, limitations and best practices. It was considered by some to serve as a of refresher training. One NGO participant elaborated,

*“I believe it is useful for HEWs to develop themselves/their skills on disease identification, treatment, and house to house visit for mothers after birth. It also improves the CBNC programme in my opinion.”* (NGO-Oromia)

#### Challenges in organizing performance review and clinical mentoring meetings for the CBNC programme service providers

##### Financial

A majority of the participants indicated lack of funding to hold regular performance review and clinical mentoring meetings. In contrast a few indicated that funding was not a challenge as it was financially supported by NGO partners.

##### Frequency of meeting

Participants reported different frequencies for the meetings that had taken place. While some said it took place biannually according to schedule, a similar number said that there had not been a performance review and clinical mentoring meeting since the CBNC programme training. A few said the meeting was annual or that there was no specific schedule. Finding a mutually convenient time for all participants was also said to be challenging.

##### Human resource

Given the insufficient number of district health office staff trained in the CBNC programme, participants also said that there was a shortage of staff to hold these meetings.

### Medicines and essential supplies

#### Mechanisms for acquiring and distributing medicines and supplies for the CBNC programme

The CBNC programme was implemented with both short- and long-term strategies for medicines and supplies. Partner NGOs initially funded, procured, and distributed medicines and supplies in order to assist the Federal Ministry of Health, the Pharmaceuticals Fund and Supply Agency and the Regional Health Bureaux. With programme maturity, CBNC medicines and supplies were planned to be incorporated into the Integrated Pharmaceutical Logistics System. The Pharmaceuticals Fund and Supply Agency would then be responsible for forecasting, procuring, and delivering medicines and supplies to health centres. Additionally, this agency would provide training on Integrated Pharmaceutical Logistics System and associated supply related tools (e.g. bin cards and Health Post Monthly Report and Resupply forms) for health centres and health post staff.

Interviewees were asked about the CBNC programme specific antibiotics (amoxicillin and gentamicin) and supplies including registers, chart booklets (the algorithm for the management of illness in young infants), referral forms, weighing scales and breathing timers. At the time of the interview, many reported that supplies were sufficient. Study participants indicated that partner NGOs distributed a *starter kit* to HEWs during the CBNC programme training. A participant from an NGO pointed out,

*“All these items are distributed during the training. We have a table prepared to register the list of materials distributed for each HEW … They sign on the sheet to confirm that they have received the items.”* (NGO – Amhara)

To re-supply health posts, health centres obtained gentamicin and amoxicillin from the district health offices. They were then distributed to health posts. Nearly all trained health centre staff and a few other officials from district health offices and partner NGOs described the use of request forms for resupplying medicines from the district health office. A soft copy of the request form was provided to health centres so they could print it as needed. Participants indicated more than one option for requesting and providing needed items. One NGO staff shared,

*“There is also ongoing provision during supervision and (performance) review meetings”* (NGO – Tigray)

Nearly half of the study participants expressed their satisfaction with the acquisition and distribution of medicines and supplies and indicated no challenges regarding availability of amoxicillin and gentamicin. Some, however, faced several challenges.

#### Challenges in the acquisition and distribution of medicines and supplies for the CBNC programme

##### Supply management problems at health posts

Lack of proper tracking and identification of out-of-stock medicines delayed the timely requests by HEWs, which ultimately resulted in stock-outs of amoxicillin and gentamicin. HEWs did not always use bin cards for commodity management due to a combination of negligence and lack of training.

*“This training (Integrated Pharmaceutical Logistics System-IPLS) has never been given separately. It has been given along with other trainings such as Performance Review and Clinical Mentoring Meeting, Integrated Refresher Training and other similar trainings. Most of the staff are not trained on IPLS. I don’t think any of the HEWs have got this training.”* (NGO – SNNP)

##### Supply side problems

Supply side challenges included unavailability of gentamicin in the right dosage for health post use (20 mg/2 ml). At the time of the study, this dose of gentamicin was not included in the country’s essential drug list.

*“There was some shortage for gentamycin last year and even this medicine is not available at PFSA (Pharmaceuticals Fund and Supply Agency) and they are under the procurement process. But there is some delay in the procurement process.”* (Health centre staff – Amhara)

Medicines were sometimes distributed very close to their expiration date, limiting their utility.

Almost one-third of the study participants indicated that insufficient timers for counting breathing rates were distributed to HEWs at the end of their training. They also reported that some HEWs were not fully comfortable with the weighing scales distributed and considered them inconvenient and risky to use.

##### Transportation challenges

Most participants mentioned transportation as another big challenge in the distribution of medicines and supplies because some health posts were very remote or inaccessible.

### Information: reporting

#### Mechanisms for reporting the CBNC programme data

Monitoring the CBNC programme required active reporting between all levels of the district health system on the coverage of care across the continuum including antenatal care, institutional delivery, and postnatal care, as well as information on possible serious bacterial infection treatment in young infants. More than half of study participants stated that a monthly report was compiled at health posts and sent to health centres. Staff at the health centres aggregated the data, ran a quality assessment check on the reports, made necessary changes, added a cover letter and sent it to the district health office. From the district, the reports were then submitted to the zone on a quarterly basis.

#### Challenges in reporting the CBNC programme data

##### Report format limitations

At the time of the study, the health management information system reporting format did not capture information on the management of possible serious bacterial cases at health posts.

*I think there is some limitation in the reporting format. The format simply asks the number of newborn children who were identified and the number of possible serious bacterial infection cases. There is no section to include the number of cases treated at the health post level, the number of referral cases, number of children who completed a full dose of medication and about the outcome of the treatment.”* (Health centre staff – Amhara)

Rather, this information was either collected from health post registers during supervisory visits using a reporting format prepared by NGOs or collected at biannual performance review and clinical mentoring meetings.

##### Poor quality reports

While many believed that the CBNC programme related information from monthly reports could be used to plan for and make informed decision on training, supply of medicines, budgeting and creating demand for CBNC programme services, only a few participants expressed their satisfaction with the quality of the reports. Insufficient training of health centre staff and HEWs contributed to the poor-quality data. Furthermore, due to a combination of negligence and high workload on HEWs, incomplete data were often recorded for maternal and newborn health related indicators.

*“There are quality problems with the reports. Sometimes they over report and when we call them, they see the register again and tell us the correct figure.”* (District health office staff – Oromia)

### Service delivery

#### Mechanisms for CBNC programme referrals and back-referrals

When identifying young infants with possible serious bacterial infection, HEWs were expected, as a first step, to refer the mother and child to the health centre after providing a pre-referral dose of gentamicin and amoxicillin. As such the CBNC programme delivery relied on an active referral and back referral system between health posts and health centres. Printed copies of the referral forms were given to the HEWs from health centres during their regular monthly meetings. HEWs were trained to use these forms for referrals, which included information on the name of the child and caretaker, age, sex, temperature, weight, type of illness, and name and dose of medicine(s) given as a pre-referral treatment, especially in cases of possible serious bacterial infections. This form was given to the mother to take to the health centre, along with the young infant. Similarly, after receiving treatment, health centre staff were expected to use a back-referral form to fill in the information regarding the status and treatment of the child for the health post to follow-up.

#### Challenges in CBNC programme referrals and back referrals

##### Weak referral system

Most participants felt the referral system was weak, citing examples of infrequent use of and incomplete information on the referral forms. Additionally, in most cases, referral follow-up was also criticized as many health centres did not use back referral forms, thus leaving HEWs with no information regarding the diagnosis, treatment and follow-up treatment once the child was sent back to health post.

*“We have never given feedback yet. This is not related to any challenge. It is just not a common practice here. For example, when we refer to a hospital, there is no feedback that we get from the hospital too. This is what we are used to”* (CBNC-trained health centre staff – SNNP)

Study participants also acknowledged the importance of back referrals.

*“I think we have to start the back-referral system strongly considering the possibility of discontinuing treatment for newborn children”* (Health centre staff – SNNP)

Lack of electricity for making copies of referral forms was also a challenge pointed out by one health centre and one NGO staff.

##### Lack of community awareness on the importance of referrals

Many participants agreed that children with possible serious bacterial infections were referred to the nearest health centre, however, over a half of the study participants concurred that the referral process was challenging, as many mothers resist taking their children to the health centre for reasons that include lack of transportation and finances, lack of proper communication between HEWs and parents, and poor health-seeking behaviour.

*“Most parents refrain from taking their child to the referred health centre due to a perception that a woman should not go out of the house after giving birth”* (NGO staff – Tigray)

### District-level ownership and governance for the CBNC programme

#### Mechanism for transfer of ownership for the CBNC programme to the district health office

The CBNC programme was introduced with a guiding principle that after the initial phase of NGO support, government at national, regional, district and kebele levels would take the lead in planning, resourcing, implementing, and monitoring the programme. Regional and zonal offices were expected to coordinate and take guidance from partner NGOs for proper implementation of the CBNC programme.

*“The government, i.e. public sector, is the main actor and owner of every programme including CBNC/ICCM (Integrated Community Case Managment) … Our responsibility is to support them, improving skills gap and giving them direction in supportive supervision. As partners, we only support them to carry out their activities appropriately. We do not replace them in any activity from planning up to report … .They take the lead in leading the training as well. Through this process, we are creating a sense of ownership in them”* (NGO-SNNP)

In addition, the sense of ownership by the district level team responsible for overseeing the CBNC programme was considered vital for integrating the services into the district health delivery system.

There was a varied sense of ownership for the CBNC programme. Four participants from district health offices and one from a health centre said that the CBNC programme had sufficient ownership.

*“There is a sense of ownership for the program from their (district health offices) side but there is a need for joint planning and strong collaboration in a continuous manner.”* (Health centre staff – SNNP)

A few health centres and NGO participants, however, indicated the lack of ownership by district health offices.

#### Challenges of district level ownership for the CBNC programme

##### Insufficient human resource

One participant said there was only one person overseeing, organising and reporting the CBNC programme activities at the district health office, which was said to be insufficient. Another shared that the appropriate person to manage the CBNC programme was not trained at the district level.

*“If heads of district health offices get the training their attention for CBNC will be higher”* (District health office staff – Amhara)

In addition to the district level ownership, health centre level ownership was considered important. Only one or two health centre staff members were trained rather than all staff treating under-5 children or providing support to HEWs at health posts. Participants said that if more of the relevant staff were trained, there would be an improved sense of ownership across all levels.

##### Insufficient district level involvement in the CBNC programme implementation

A few study participants from health centres and district health offices expressed concerns that the CBNC programme was not using the district’s own mechanisms for requesting and distributing medicines and supplies. Rather it depended on NGO partners. This raised their concerns regarding the sustainability and future government ownership of the CBNC programme.

*“There is a need for a joint planning exercise between health facilities, the district office and supporting NGOs. Unless we start to work jointly starting from now CBNC activity may fail to be sustained when the support of the NGO is interrupted sometime in the future.”* (Health centre – SNNP)

##### Insufficient support from kebele administration

Ownership of the CBNC programme at the kebele level was also considered important. Only one health centre staff said that kebele administration supports them in planning and setting a direction for community action plan whereas few others pointed out that the CBNC programme was not included in kebele administration activities.

### Recommendations

**Fig. 3:**
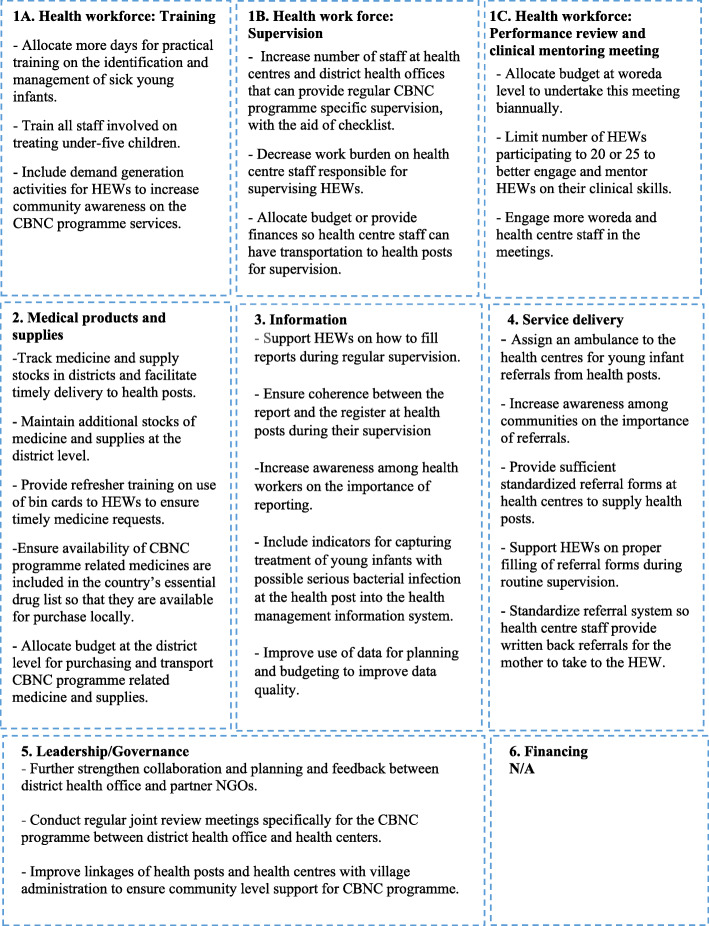
District health managers recommendations to improve the Community-Based Newborn Care programme implementation, structured by the World Health Organization health system building blocks

Study participants were asked to provide recommendations to improve the implementation of the CBNC programme. Their responses are presented in Fig. 3.

## Discussion

Despite skills training on a national scale for HEWs on the management of possible serious bacterial infections in young infants, health system essentials to provide antibiotics were not in place 20 months after the start of the service. These health system essentials included: medicines and supplies; standards, processes and human resources for supervision and clinical mentoring; standards and process for referrals; and health information on treatment and referral of young infants. In turn, these deficits affected leadership and governance at the district level.

For sick young infants, injectable antibiotics at community level can save lives [20]. Indicative of a positive government commitment to address neonatal mortality, HEWs' training on the management of possible serious bacterial infection in young infants was scaled-up to most of the country faster than initially planned [19]. However, the CBNC programme training was considered to be of low intensity by health centre staff and district health managers. Indeed there is some evidence that HEWs were not treating young infants with possible severe bacterial infection according to national guidelines [21]. Further, studies have shown that training alone has little effect in improving health care provider performance [22]. Strategies that include training in combination with supportive supervision and ongoing clinical mentorship are more likely to yield better outcomes [22, 23]. However, in this study, trained managers able to provide supervision and mentorship were not consistently available in some district health offices and health centres. Where trained staff where available, the content of their training was reported to be inadequate to manage and provide standardized and CBNC programme specific support for HEWs. Furthermore, performance review and clinical mentoring meetings were either not held after the CBNC programme training or were held irregularly. Sufficient district level managers and health centre staff with pre-service training on how to provide supportive supervision and clinical mentorship can improve the performance of CBNC programme service providers.

Community health workers can better manage sick children when provided with regular supply of essential medicines [24–26]. To initiate the CBNC programme, HEWs were provided with a starter kit of antibiotics to cover 12 months, which was procured and distributed by partner NGOs. This was a necessary alternative to expedite the start of the programme. Replenishment for the following year was also procured and distributed by implementing partners and UNICEF; amoxicillin dispersible tablets and gentamicin started being processed through the national system only at the end of 2017 [27]. This indicates that CBNC was scaled-up relying on similar support from implementing NGOs, rather than the district’s system for procuring and supplying medicines. This has resulted in periodic shortages of antibiotics for the CBNC programme at health posts [28]. Stock-outs of supplies and medicines is associated with a low demand for newborn services in communities [29]. Health systems need to build the capacity of the supply chain for essential drugs at both district and national level prior to scaling-up new health services.

The WHO guidelines for the management of possible severe bacterial infection through community health workers highlights the need to include strong monitoring and evaluation into the implementation processes [30]. It recommends that the diagnosis, treatment and response to treatment need to be monitored and coverage tracked in order to provide lessons for service improvement. The routine district level health information system for the CBNC programme did not collect data on the diagnosis and treatment of young infants with possible serious bacterial infection at the health post level 20 months after the service was initiated. Where these indicators were available, they were collected through a parallel reporting system. As good decision making requires data of sound quality, it is important for key indicators of a new service to be included in the routine health information system at the time of service initiation [31].

The CBNC programme relied on an active referral and back referral system between health posts and health centres. The referral system in this study was said to be weak. Other studies investigating the CBNC referral system also found insufficient use of referral slips by HEWs when referring to health centres and back referrals from health centres to health posts indicating poor follow-up for highly vulnerable sick young infants once they returned home [32, 33]. As reported in this study, caregivers also had suboptimal follow-up on their referral, perhaps influenced by the absence of a referral slip, which helps caretakers understand and adhere to the provided referral [33]. District health managers need to ensure that the referral linkages between the different levels of the primary health care system are strengthened, ensuring the availably and use of referral and back referral slips at each level.

Government ownership and leadership is needed for successful implementation and scale-up of health services [34]. The heavy district-level involvement of partner NGOs in implementing the CBNC programme illustrates the tension between the short-term and long-term planning for the service. To ensure the timely launch of the CBNC programme, NGOs were involved in the training and supervision of health workers and health managers, procurement and distribution of antibiotics and collecting service utilization data. This task shifting from the district health office to NGOs was necessary as a short-term strategy. However, in the long term, it deterred the district level health system from taking full ownership and governance for the effective planning and management of the service. NGO support is usually dependent on external funding and is available for a limited amount of time. Government-NGO partnerships should build in a transition period as early as possible where tasks and skills needed for the management of a health service are fully transferred to the government system.

These findings need to be interpreted within the scope of the study aim. The challenges highlighted are timebound; they focus on the operational and capacity barriers faced 20 months after a new health service for newborns was initiated. This study excluded health care financing as financial allocation for the CBNC programme was dealt with at national level through support form external sources such as UNICEF, rather than at the district level [18]. Further, as the focus of this study was the challenges faced by managers when implementing and streamlining the CBNC programme, challenges at the point of service delivery by health workers were not included. Similar assessments are needed to understand the nature of these and other challenges, including finance and service delivery at the provider level, when the service is further embedded into the health system.

## Conclusions

This study provided an in-depth assessment of how a new health service for newborns was integrated into an existing district health system and identified key barriers to its integration, 20 months after service initiation. Several health system challenges were identified that indicated the importance of having pre-defined and tested standards in place for supervision, health worker training, the routine health information system and for the drug supply chain. Our findings also showed how working with NGO partners can achieve rapid change but has implications for the long-term ownership of the programme. Collaborations between government and NGOs partners need to, early in the implementation period, identify and fill gaps in the district health system that ensure the longevity of the service once the NGO support is withdrawn. The findings from this study have relevance for low-and-middle income countries that are planning to integrate new health services into the district health system.

## Supplementary Information

**Additional file 1.** Qualitative Tool.

**Additional file 2.** A priori and emerging codes and sub-codes used for analysing interviews conducted with district level health managers and implementing of the Community-Based Newborn Care (CBNC) programme, using the World Health Organization (WHO) health system building block framework.

## Data Availability

The qualitative data generated and analysed in this study are not publicly available due to issues of confidentiality and privacy. Even though the respondents’ names have been removed from the transcripts, they do include names of facilities and districts mentioned by respondents that could lead to their identification.

## Abbreviations

ANC: Antenatal care
CBNC: Community-Based Newborn Care
HEW: Health extension worker
ICCM: Integracted Communicty Case Managment
IPLS: Integrated Pharmaceutical Logistics System
NGO: Non-governmental organization
PFSA: Pharmaceuticals Fund and Supply Agency
SNNP: Southern Nations, Nationalities and Peoples
UNICEF: United Nation Children’s Fund
WHO: World Health Organization
