# Supplementary material for: District health managers perspectives of introducing a new service: a qualitative study of the community-based newborn care programme in Ethiopia
Source: BMC Health Serv Res. 2021 Aug 9;21:783. doi: 10.1186/s12913-021-06792-8 (PMC8351343; doi:10.1186/s12913-021-06792-8)
Supplement: Supplementary file 1 — Additional file 1. Qualitative Tool. [file 12913_2021_6792_MOESM1_ESM.pdf]

Additional file 1. Qualitative Tool

In Depth Interview with Woreda Health Office, Health Centre and CBNC Implementing NGO Staff

s.no	MODULE	RESPONSE
**1.**	**Background on woreda and socio-demographic information in interviewee**	
Q1.1	Date<br>(**Gregorian Calendar**)	|___|___| / |___|___| / |___|___| dd / mm / yy
Q1.2	Region Code	|___|___|
Q1.3	Zone Code	|___|___|
Q1.4	Woreda name	
Q1.5	Woreda code	|___|___|
Q1.6	PHCU code (if at health centre)	|___|___|___|
Q1.7	Unique ID<br>(Composed of the 2 digit zonal, 2 digit woreda code, 2 PHCU code)	**|___|___|/ |__|__|/ |___|___|**<br>**Zone / Woreda / PHCU**
Q1.8	Interviewer Initials	|___|___|
Q1.9	Type of Respondent	**1 = Woreda Health Office**<br>**2 = Health Centre**<br>**3 = NGO**
Q1.10	Gender	**1= Male 2= Female**
Q1.11	Did you read the staff member the consent form?	**1 = Yes 2 = No**
Q1.12	Did the staff member agree to be interviewed?	**1 = Yes**<br>**2 = No (Skip to Q1.14)**
Q1.13	***If not,*** *why not?*	____________________<br>***END***
Q1.14	IDI start time	**|___|___|:|___|___|**
Q1.15	IDI end time	**|___|___|:|___|___|**
**Interviewer*: Thank you for agreeing to be interviewed. I would first like to ask you some questions about your role in this organization.***		
Q1.16	What is the current position you hold in the organization?	
Q1.17	What is the length of time you have spent in this position?	
Q1.18	What is the highest educational qualification you have obtained?	

Q1.19	Have you been responsible for overseeing the CBNC program for at least the last 3 months?	**1 = Yes 2 = No**
Q1.20	Have you received training in CBNC?	**1 = Yes**<br>**2= No If no go to Theme 3 (Drugs)**

No.	Modules	Questions	Responses	Probe
Q2	**Training**	***Now I would like to ask you some questions about the CBNC training.***		
Q2.1		What did the CBNC training cover?		*On diagnosis, treatment, supervision, reporting*
Q2.2		What were the good aspects of this training?		What skills or knowledge have been improved
Q2.3		What aspects of the training can be improved?		*Number trained, content, number of days, follow up*
Q2.4		In your opinion, in addition to you, who else in the woreda should get CBNC training in order to effectively run the program?		**If NGO** ask who else in the organization rather than the woreda
Q3	**Drugs**	***Now in this section I would like to ask you about the supply chain of the drugs. First I would like to ask how you get the supply of the drugs then how you distribute them.***		
Q3.1		Can you tell me how you get Amoxicillin supply? And how you get gentamycin supply? And how you get tetracycline eye ointment supply?		*Ask separately for supply on each drug*<br><br>*Frequency of supply*
Q3.2		What are challenges of receiving each of these drugs?		*Ask separately for supply on each drug*
Q3.3		Can you suggest how the supply of each these drugs to the woreda can be improved?		***if at health centre** ask for health centre*<br>*Ask separately for supply on each drug*
Q3.4		Can you tell me about the Amoxicillin distribution? And gentamycin distribution? And tetracycline eye ointment distribution?		*Ask separately for distribution on each drug Frequency of distribution*
Q3.5		What are the challenges of timely distribution of each of these drugs to health post level?		*Ask separately for distribution on each drug*<br><br>*Financial and logistical challenges*

Q3.6		How can the timely distribution of each of these drugs from woreda be improved?		*Ask separately for supply on each drug*
Q4	**Supplies**	***Now in this section I would like to ask you about supplies. First I would like to ask how you get and the distribute forms and registers.***		
Q4.1		Can you tell me about how you get chart-booklets? And how you get VSD referral forms? And how you get 0-2 month registration books?		*Ask separately for each item*<br><br>*Frequency of supply*
Q4.2		What are challenges of receiving each of these supplies?		*Ask separately for each item*
Q4.3		Can you suggest how the supply of these items to the woreda can be improved?		*Ask separately for each item*
Q4.4		Can you tell me about the chart-booklet distribution? And VSD referral forms distribution? And 0-2 month registration books distribution?		*Ask separately for each item*<br><br>*Frequency of distribution*
Q4.5		What are the challenges of timely distribution of each of these items to health post level?		*Ask separately for each item*<br><br>*Financial and logistical challenges*
Q4.6		How can the timely distribution of each of these items from woreda be improved?		*Ask separately for each item*<br>*Financial and logistical challenges*
Q5	**CBNC Supervision**	***Now I would like to ask you about the supervision that you provide.***		
Q5.1		Can you tell us how CBNC related supervision from woreda to health centre takes place?		***If interviewing health centre or NGO staff*** *ask supervision to health post*<br><br>*Is there a standard checklist available?*

				How is the checklist used?<br><br>*Frequency, type of staff who supervise*<br>*How feedback is provided*
Q5.2		How do you assess that the skills of the health centre staff are appropriate to guide the HEWs in the treatment of VSD		***If interviewing health centre or NGO staff*** *then ask "how do you assess the skills of the HEWs in the treatment of VSD?"*<br><br>*Monthly performance review meeting*
Q5.3		What are the challenges to CBNC related supportive supervision?		*Distance, sufficient human resources*
Q5.4		How can CBNC related supervision be improved?		
Q6	**Performa nce Review and Refresher Training Meeting-PRRT**	***Now I would like to ask you about the PRRT conducted at this woreda***		***PRRT is a meeting conducted once or twice a year with HEWs, HC staff and NGO to review work on CBNC/ICCM***
Q6.1		Can you tell us if a PRRT meeting was held in this woreda?		
Q6.2		**If yes,** can you tell us how the PRRT meeting is organized?		*Frequency, participants, planning, location*
Q6.3		Can you go through with us step by step what happens in a PPRT meeting?		*How are the skills of the HEWs being refreshed or improved?*
Q6.4		What are the challenges in the logistics and conduct of the PRRT meetings?		
Q6.5		How can the PRRT for CBNC be improved?		*Content, participant, frequency*

Q7		***Now I would like to ask you about CBNC reporting in this woreda***		
Q7.1		Can you describe the reporting on Very Severe Disease from health centre to woreda?		***For health centre***<br>*From health post to health centre*<br><br>***For NGO***<br>*Content of the report they prepare (identification of cases, referral, treatment, completion)*<br><br>*Frequency of reporting*
Q7.2		Can you tell me how is the data is compiled and transferred to the zone level?		***If health centre*** *ask how data are compiled and transferred to the woreda?*<br><br>***If NGO***<br>*How it is transferred upwards*
Q7.3		Can you tell us about the quality (accuracy and completeness) of the data that you receive from the health centre?		***If health centre*** *ask about the quality of the data they received from health post?*<br>***IF NGO***<br>*Quality of data that they receive from whichever level*<br>***If there is a problem*** *at what is the level (HP or HC) or source of the problem?*
Q7.4		Can you tell us how the quality (accuracy and completeness) and reporting of the VSD related information can be improved?		*Quality and utilization*
Q7.5		Can you tell me how the data on VSD is being utilized at the woreda?		***If health centre*** *how data is being used at the health centre*<br><br>***If NGO*** *how data is being by the NGO*

				Data usage for planning/budgeting
Q7.6		Can you tell us how the utility of the data at the woreda can be improved?		***If health centre** how data utility can be improved at the health centre*<br><br>***If NGO** how the data utility can be improved for NGO*
Q8	**Linkages between NGO and Health System**	***Now I would like to ask you some questions on the linkages between the woreda health system and CBNC implementing NGO***		
Q8.1		Can you describe the operational linkages between the woreda health office and the CBNC implementing NGO?		***IF health centre:***<br>*Ask about operational linkages between health centre and NGO*<br><br>*Frequency of meeting*<br>*Planning together*<br>*Trainings*<br>*Supplies*<br>*Supervisions*<br>*Performance review*<br>*Reporting*
Q8.2		Can you describe how the operational linkages can be improved?		*Ownership,*<br>*For all listed from Q8.1*
Q8.3		What is the operational linkages between the woreda and the health centre?		
Q8.4		Can you describe how the operational linkages can be improved?		
Q9	**Referral**	***Now I would like to ask you questions on referral between the health post and health centre***		
Q9.1		Can you describe how referral system from health post to health centre works for VSD for children under 2 months?		*Use of forms for referral*
Q9.2		Can you suggest on how the referral system can be improved?		

Q9.3		Can you describe how the back referral from health centre to health post for children under 2 months with VSD works?		*Use of back referral forms*
Q9.4		Can you suggest how to develop/improve on the back referral system?		
Q10				
Q10.1		Do you have any comment on how to improve the overall VSD treatment in this woreda?		
