# Supplementary material for: District health managers perspectives of introducing a new service: a qualitative study of the community-based newborn care programme in Ethiopia
Source: BMC Health Serv Res. 2021 Aug 9;21:783. doi: 10.1186/s12913-021-06792-8 (PMC8351343; doi:10.1186/s12913-021-06792-8)
Supplement: Supplementary file 2 — Additional file 2. A priori and emerging codes and sub-codes used for analysing interviews conducted with district level health managers and implementing of the Community-Based Newborn Care (CBNC) programme, using the World Health Organization (WHO) health system building block framework. [file 12913_2021_6792_MOESM2_ESM.pdf]

Addental file 2. A priori and emerging codes and sub-codes used for analysing interviews conducted with district level health managers and implementing of the Community-Based Newborn Care (CBNC) programme, using the World Health Organization (WHO) health system building block framework.

WHO Building Blocks	Code	Sub-code
**1A. Health workforce:** Training	Process for training	Length of training
		Training participants
		Content of training
		Value of training
		Post training follow up
	Challenges of training	Length of training
		Training participants
		Content of training
		Training follow-up
		Ownership and governance
	Ways to improvement	Length of training
		Training participants
		Content of training
		Ownership and governance
		Follow-up training
**1B. Health workforce:** Supervision	Mechanisms	Content of supervision
		Checklist for supervision
		Frequency of visits
		Provider of supervision
		Feedback mechanism
	Challenges of supervision	Supervision
		CBNC specific supervision
		Financial
		Transport
		Human resource
		Ownership and governance
	Ways to improve	Human resource
		Financial
		Transport
		Content of supervision
		Frequency of supervision
		Ownership and governance
**1C. Health workforce:** Performance review and clinical mentoring meeting (PRCMM)	Mechanisms	Frequency of PRCMM
		Length and location of PRCMM
		Participants of PRCMM
		Content of PRCMM
		Financial
		Technical/logistical
	Challenges to PRCMM	Frequency and length of meeting
		Transportation
		Human resource
		Ownership and governance
		Financial
	Ways to improve PRCMM	Frequency
		Length
		Ownership and governance
		Participants
		Content

**2. Medicines supplies and products**: CBNC medicines, materials, and supplies	Process for acquiring and distribution	Starter kit given after training
		Post training distribution
		Source of distributor
		Standard mechanism for distribution for non-CBNC medicines
	Frequency of distribution	
	Challenges	Transportation
		Availability
		Financial
		Ownership and governance
	Ways to improve	Management of supply chain
		Training
		Availability
		Financial
		Ownership and governance
**3. Information:** CBNC reporting	Mechanisms	Content of report
		Flow of report
		NGO role in reporting
		Frequency of PRCMM report
		Quality
	Challenges	Format of reporting
		Format of reporting
		Ownership and governance
		Human resource
	Ways to improve reporting	Training on reporting
		Content of data
		Use of data
		Quality of data
		Frequency of reporting
**4. Service delivery:** Referral	Mechanisms	Flow of referral
		Use of Forms
	Challenges	Distance
		Referral system
		Forms
		Ownership and governance
		Referral follow-up
	Ways to improve	Referral system
		Forms
		Supervision and Training
		Referral follow-up
		Transportation
